# Exploring Bioactivities and Peptide Content of Body Mucus from the Lusitanian Toadfish *Halobatrachus didactylus*

**DOI:** 10.3390/molecules28186458

**Published:** 2023-09-06

**Authors:** Marta Fernandez Cunha, Ezequiel R. Coscueta, María Emilia Brassesco, Rita Marques, José Neto, Frederico Almada, David Gonçalves, Manuela Pintado

**Affiliations:** 1 CBQF—Centro de Biotecnologia e Química Fina—Laboratório Associado, Escola Superior de Biotecnologia, Universidade Católica Portuguesa, Rua Diogo Botelho 1327, 4169-005 Porto, Portugal; mfcunha@ucp.pt (M.F.C.); mbrassesco@ucp.pt (M.E.B.); mpintado@ucp.pt (M.P.); 2 MARE—Marine and Environmental Sciences Centre, ISPA Instituto Universitário de Ciências Psicológicas, Sociais e da Vida, Rua Jardim do Tabaco, 34, 1149-041 Lisbon, Portugalfrederico.almada@gmail.com (F.A.); 3 Institute of Science and Environment, University of Saint Joseph, Rua de Londres 106, Macau SAR, China; david.goncalves@usj.edu.mo

**Keywords:** ichthyocrinotoxins, antimicrobial, antioxidant, antihypertensive, peptides

## Abstract

Identifying bioactive molecules from marine organisms is still vastly understudied. Fish remain an untapped source of bioactive molecules, even when considering species whose toxicity to other fish species has been noticed before. We assessed potential applications of crude body mucus of the Lusitanian toadfish (*Halobratachus didactylus*) and characterized its peptide fraction composition. Mucus samples from three individuals (two wild and one captive) revealed potential antioxidant, antihypertensive, and antimicrobial activities. For antioxidant activity, the best results of 2371 ± 97 µmol Trolox Equivalent/g protein for ORAC and 154 ± 6 µmol Trolox Equivalent/g protein for ABTS were obtained. For antihypertensive activity, the relevant inhibitory activity of ACE resulted in IC_50_ of 60 ± 7 µg protein/mL. Antimicrobial activity was also identified against the pathogenic bacteria *Escherichia coli* and *Listeria monocytogenes*. The peptide profile of the crude body mucus was obtained through size exclusion chromatography, with a conspicuous peak at ca. 800 Da. LC-MS/MS allowed the detection of the most probable peptide sequences of this dominant peptide. This is the first study where the bioactive potential of mucus from the Lusitanian toadfish is demonstrated. Peptides with such properties can be applied in the food and pharmaceutical industries.

## 1. Introduction

Multidrug-resistant bacteria pose a significant challenge for research on potential pharmacological agents. In recent years, little progress has been made in addressing this issue with fewer new effective molecules [1]. Additionally, chronic diseases such as diabetes, cardiovascular disease, cancer, and psychotic disorders have also demonstrated, in some patients, multidrug resistance, resulting in substantial societal costs and creating the need for new alternative molecules [2]. Several antimicrobial peptides derived from natural sources like plants, invertebrates, and mammals have demonstrated value, but only a few have been launched in the market [1,3]. The food industry also seeks high-quality and safer bioactive molecules to be used as preservatives or health enhancers, and nature is a rich source of new peptides with diverse bioactive potentials, including antimicrobial, antioxidant, and antihypertensive potential [4]. In contrast to their terrestrial counterparts, marine organisms represent a largely untapped potential for drug discovery. This represents a striking difference considering their substantial contribution to the Earth’s biodiversity, with oceans covering 70% of the planet [5,6].

Despite abundant marine biodiversity, researchers have not extensively explored marine organisms for drug discovery compared to plants, primarily due to historical challenges, such as natural constraints to access marine habitats and species [6]. Nevertheless, marine organisms thrive in harsh and challenging environments, making them valuable reservoirs of bioactive substances [7,8]. Fish are the most diverse group of vertebrates, occurring virtually across all aquatic habitats. They possess robust immune systems that have to tackle numerous pathogens [9]. The body mucus plays a crucial role in fish immunity as it acts as a protective barrier against the surrounding environment, serving as a first line of defense [9,10]. Fish secrete ichthyocrinotoxins composed of various immune-related molecules within the mucus, including C-reactive proteins, lysozymes, immunoglobulins, proteolytic enzymes, and antimicrobial peptides [10,11]. Although researchers have extensively studied fish mucus to evaluate immunity, most studies focus on fish in aquaculture systems, which face a higher risk of bacterial infections due to confinement. In turn, frequent treatments lead to the emergence of multidrug-resistant bacteria [12]. To address this concern, recent publications within the past six years have explored methods to enhance fish immunity by incorporating polyphenols, plant extracts, and probiotics into their diets [13,14,15]. When assessing immune parameters in fish, researchers prefer mucus collection over blood due to its less invasive nature [16]. *Sparus aurata*, *Oncorhynchus mykiss*, and *Salmo salar* have undergone extensive studies regarding mucus composition due to their prevalence in human diets and widespread farming in aquaculture systems. However, only a few studies have investigated the presence of novel bioactive molecules within fish mucus, resulting in a limited number of described antimicrobial peptides (AMPs), such as NK-lysin, CF-14, and hepcidin type 2-like AMPs from *Salmo salar*, catfish and *Takifugu pardalis*, respectively, which exhibited potent antibacterial effects against Gram-positive and Gram-negative fish bacteria strains [17,18,19].

To date, only the protein activity of a known western Atlantic venomous species from the family Batrachoididae (*Thalassophryne nattereri*) has been described [20,21]. Ichthyocrinotoxins have been reported in another western Atlantic species (Oyster toadfish—*Opsanus tau*) [22] but lack additional characterization.

The eastern Atlantic and Mediterranean Lusitanian toadfish, *Halobatrachus didactylus*, is found in estuaries along the west coast of Portugal [23]. Males of this species display parental care of eggs and larvae, suggesting a possible ecological role for mucus in protecting eggs [24]. Moreover, when held in captivity alongside other fish species, *H. didactylus* often causes the rapid death of other fish (personal observations). Our investigation aims to identify novel bioactive molecules within the mucus of *H. didactylus*, particularly peptides. While the existing literature has primarily focused on the ecology and ethology of this species, our study represents the first bioprospection of its body mucus pharmacological applications.

## 2. Results and Discussion

### 2.1. Peptide Profile

Body mucus was collected from three specimens of *H. didactylus*: one captive (HdTAG) and two wild specimens (HdSES1 and HdSES2). Mucus was collected with a synthetic sponge that was immediately frozen and later washed with a 100 mM KPB buffer solution and centrifuged. We initially employed high-performance size exclusion chromatography (HPSEC) to compare the peptide profiles between different specimens.

**Figure 1:**
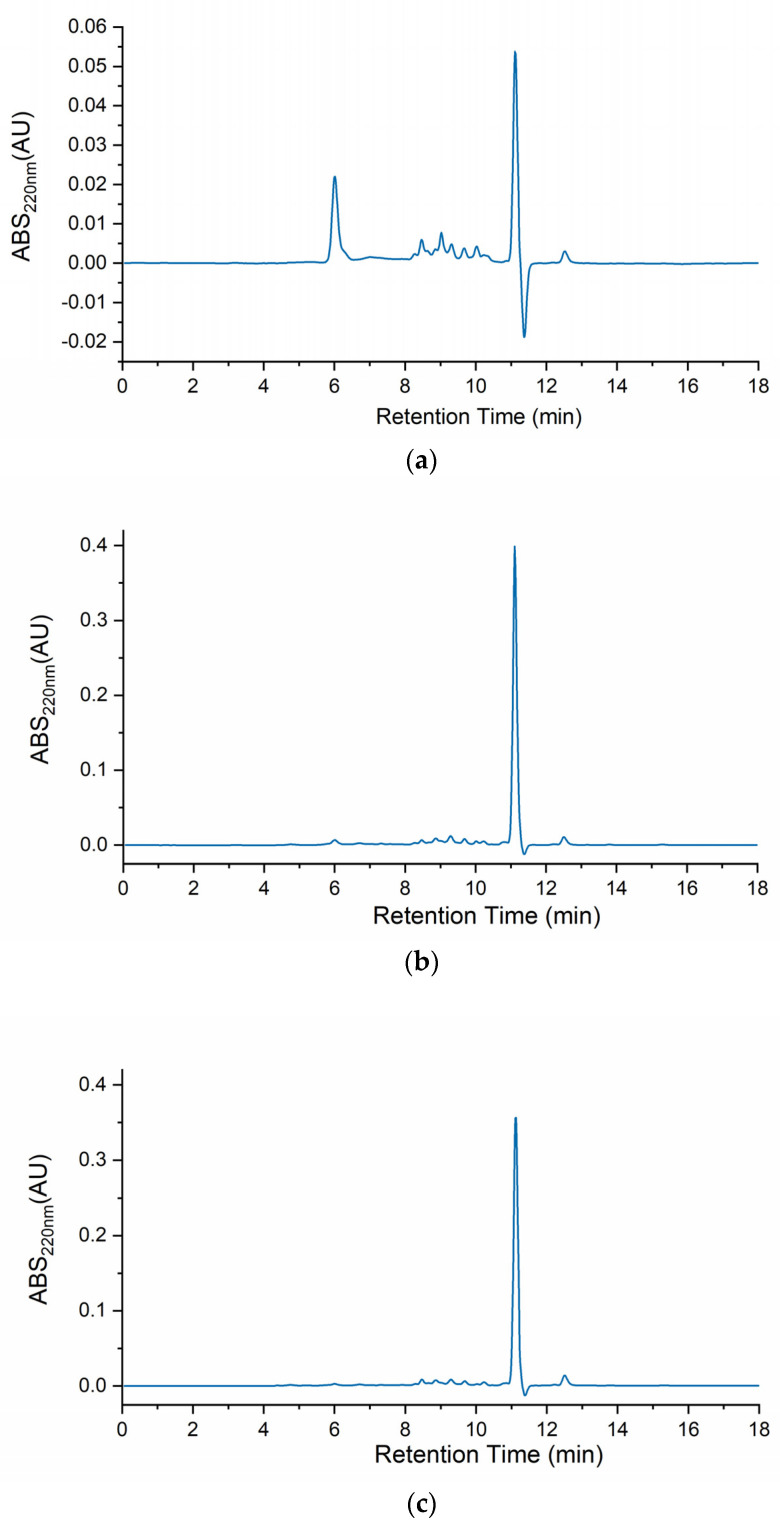
HPSEC chromatograms of the body mucus from the three *H. didactylus* individuals (**a**)—HdTAG, (**b**)—HdSES1, and (**c**)—HdSES2.

Figure 1 depicts the body mucus chromatograms of the three *H. didactylus* samples. Notably, all three chromatograms exhibited a comparable distribution of molecular sizes, ranging from ca. 20 kDa to 800 Da. A prominent peak at around 11 min retention time was consistently observed across all three chromatograms, indicating the presence of relevant and common molecules with a molecular size of approximately 800 Da. This analysis provides valuable insights into the molecular size distribution profile of *H. didactylus*, particularly highlighting the presence of putative bioactive peptides with smaller molecular sizes.

In contrast with our results, the chromatographic profile of *Opsanus tau* (Batrachoididae) revealed three distinct peaks [22]. This species was also revealed to be toxic to other fish sharing the same tank while in captivity. These results, although highlighting possible differences between body mucus protein profiles from different species, were described more than 40 years ago. Therefore, before drawing any further conclusions, a comparative analysis using identical methodologies should be performed.

A previous study has also reported the identification of a single peak through HPLC analysis using a reverse-phase column for antibacterial fractions derived from the skin mucus of *Takifugu pardalis* (Tetraodontidae) [19]. Remarkably, this fraction exhibited antibacterial activity against *Escherichia coli* D31 [19]. The findings from the *T. pardalis* research indicated the presence of antimicrobial molecules with smaller molecular sizes within the mucus. These results are consistent with ours, suggesting the potential existence of bioactive antimicrobial peptides in *H. didactylus* mucus.

### 2.2. Total Protein and Antioxidant Activity

The body mucus samples from the three individuals were evaluated, HdTAG, HdSES1, and HdSES2, for soluble protein content (by BCA) and antioxidant activities (by ORAC and ABTS). Table 1 demonstrates that the HdTAG individual (captive) exhibited a higher protein concentration than the wild individuals HdSES1 and HdSES2. Additionally, the same individual demonstrated significantly higher antioxidant activity in the ORAC assay (*p* < 0.05). Notably, the ORAC antioxidant activity results indicated a potential relationship between antioxidant activity and protein concentration for the HdTAG individual. However, this correlation was absent for the ABTS assay. One possible explanation could be that these assays measure different reactions, with the ORAC assay focusing on hydrogen’s antioxidant donation capacity for radical neutralization and the ABTS assay operating through electron transfer [25]. The body mucus of *H. didactylus* exhibited antioxidant potential, which appears to be a crucial aspect of the fish’s immune defense system. That implies that reactive oxygen species may be overpopulated in stressful conditions, which can lead to cellular damage.

Antioxidant activity of Lusitanian toadfish mucus proved to be more effective than similar studies on other fish species (e.g., mackerel-*Scomber* sp. [26]). It is important to note that while we expressed our values based on protein concentration and used crude mucus, those authors did not determine the protein concentration of mackerel mucus and performed dialysis, resulting in two mucus fractions (H > 12,000–14,000 Da and L < 12,000–14,000 Da). Nonetheless, our captive and wild individuals can be compared to the farmed and wild mackerel. Regarding ORAC activity, the mucus from wild individuals in the current study (HDSES1 and HDSES2) exhibited higher values than the fraction L (less than 12,000–14,000 Da) of wild mackerel, surpassing 60 µmol TE/g. Additionally, the captive HdTAG individual showed higher ORAC activity than the fraction L (333 µmol TE/g) and the fraction H (178 µmol TE/g) of farmed mackerel. However, HdSES1 and HdSES2 did not exceed the value of 164 µmol TE/g for the fraction H of wild mackerel. Regarding ABTS, in this study results surpassed the values observed for wild and farmed mackerel mucus fractions, ranging from 2.9 to 7 µmol TE/g. The previous study on mackerel mucus suggested that farmed mackerel experiences higher stress levels, leading to increased antioxidant activity. This finding could be relevant to justify these results as the captive HdTAG individual also showed higher antioxidant activity results.

The HPSEC analysis of body mucus revealed the presence of low-molecular-weight compounds, suggesting the potential presence of antioxidant peptides responsible for the observed antioxidant activities. The significance of antioxidant peptides has been increasingly recognized in the food and pharmaceutical sectors [4,27]. Adding preservatives to avoid oxidation and extend product shelf life is crucial [27]. Therefore, there is a growing interest in identifying safe ingredients, and antioxidant peptides hold promise. These peptides have demonstrated their ability to scavenge free radicals, regulate the activities of endogenous antioxidant enzymes, and modulate antioxidant-related signaling pathways, thus playing a vital role in preventing oxidative damage that can be detrimental to human health [27].

### 2.3. Antihypertensive Activity

To our knowledge, the antihypertensive activity of fish body mucus remains largely unexplored. However, previous studies have extensively examined the angiotensin-converting enzyme (ACE) inhibitory potential of other fish materials such as salt-cured cod skin from Atlantic cod *Gadus morhua*, skin gelatin extracts from Alaska pollack *Theragra chalcogramma,* and muscle extracts from tuna *Neothunnus macropterus* and chum salmon *Oncorhynchus keta* [28,29]. Among those, hydrolysates and peptides derived from marine organisms ACE have proved to regulate blood pressure by converting angiotensin I into angiotensin II, a vasoconstrictor [29]. In this study, the inhibitory activity of ACE (iACE) using the HdTAG sample was evaluated because it exhibited higher protein concentration and antioxidant activity in the ORAC assay. This test resulted in an IC_50_ of 60 ± 7 µg mucus protein/mL, a promising outcome blocking ACE action even at low concentrations. In a comparative scale, the body mucus sample from *H. didactylus* exhibited higher ACE inhibition than the hydrolysates from other fish species such as the Skipjack tuna *Katsuwonus pelamis* (IC_50_ 2500 µg/mL), Atlantic salmon *Salmo Salar* (IC_50_ 110 µg/mL), and Chum salmon *Oncorhynchus keta* (IC_50_ 1008 µg/mL) [30]. In general, peptides with inhibitory activity of ACE lower than 100 µg protein/mL are considered to have good potential antihypertensive activity [31,32].

The specific molecules responsible for this bioactivity remain unknown, but the predominance of the 800 Da peptide suggests that this is a promising candidate for future tests separating this fraction. Peptides can block ACE through competitive and non-competitive mechanisms. In the competitive mode, the peptide binds to the enzyme, causing inhibition, while in the non-competitive mode, the peptide–enzyme combination forms a dead-end complex [30,33]. Several ACE-blocking drugs available in the market (e.g., Captopril) have been associated with significant side effects, as reported in previous studies [30,31]. Antihypertensive peptides derived from fish could serve as an alternative, with some fish peptides already identified to have no side effects [29]. From a different perspective, these peptides can become useful in the food industry as nutraceuticals apart from their pharmaceutical applications.

### 2.4. Antimicrobial Activity

**Figure 2:**
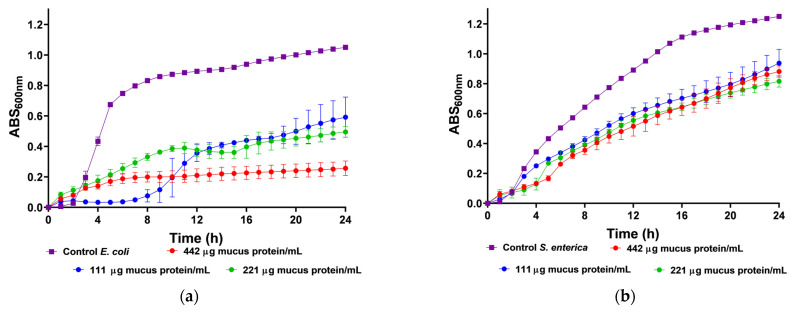

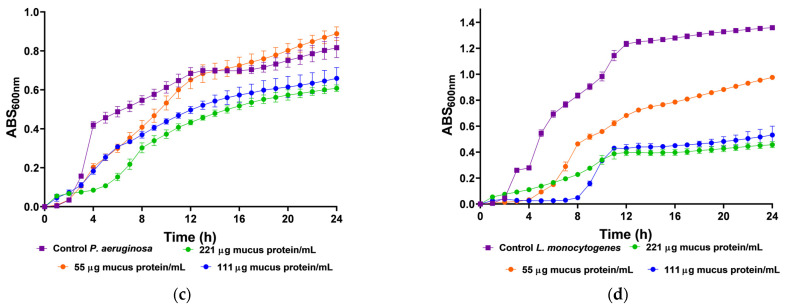
Inhibition growth curves (measured by absorbance at 600 nm) of HdTAG body mucus samples tested at concentrations between 442 and 55 µg mucus protein/mL against pathogenic bacteria; Gram-negative: (**a**)—*E. coli*, (**b**)—*S. enterica*, and (**c**)—*P. aeruginosa*; and Gram-positive: (**d**)—*L. monocytogenes*.

We utilized body mucus from the HdTAG individual to determine growth inhibition curves against pathogenic bacteria, including three Gram-negative bacteria, *Escherichia coli* ATCC 25922, *Salmonella enterica* serovar Enteritidis ATCC 13076, *Pseudomonas aeruginosa* ATCC 27853, and one Gram-positive bacteria, *Listeria monocytogenes* NCTC 10357. The HdTAG mucus sample exhibited inhibitory effects on all bacteria at concentrations ranging from 442 to 55 µg mucus protein/mL, as illustrated in Figure 2. Particularly notable were the significant reductions in *E. coli* growth by 76% at 442 µg mucus protein/mL and *L. monocytogenes* growth by 66% at 221 µg mucus protein/mL. Additionally, the HdTAG sample demonstrated reductions in *S. enterica* growth by 35% at a concentration of 442 µg mucus protein/mL and *P. aeruginosa* growth by 25% at 221 µg mucus protein/mL. While many studies on fish mucus evaluate antimicrobial activity against fish pathogenic bacteria for applications in the aquaculture industry, such as *Aeromonas hydrophila*, *Yersinia ruckeri*, *Vibrio harveyi*, and *Vibrio parahaemolyticus* [34,35,36], there are limited studies investigating the antimicrobial activity of fish mucus against human pathogenic bacteria. For instance, a glycoprotein extracted from the epidermal mucus of African sharptooth catfish *Clarias gariepinus* (CFG) displayed antimicrobial potential against *E. coli*, *L. monocytogenes*, and *P. aeruginosa* at an inhibitory concentration of 50 μg/mL of CFG [37].

Similarly, mucus derived from freshwater pool barb *Puntius sophore* demonstrated inhibitory effects upon *E. coli* and *P. aeruginosa* at concentrations ranging from 250 to 50 μg/mL of mucus [38]. These results are helpful to provide a framework for fish mucus antimicrobial activity. However, we cannot compare results directly because different authors use different methods. This study used crude venom extracts, which can be compared to the acid extraction technique used with the pool barb. This differed from the glycoprotein extraction on mucus from the African sharptooth catfish.

In comparison with another known venomous fish species, a study demonstrated a potent antimicrobial activity of the common stingray *Dasyatis pastinaca* skin mucus against the same strains of *E. coli* ATCC 25922 and *P. aeruginosa* ATCC 27853, with an inhibitory concentration of 16 µg/µL of skin mucus [39]. In this last example, the authors did not describe the mucus collection method. This finding on the ability of mucus from the body of *H. didactylus* to be antibacterial is a significant feature since there is an increase in antibiotic resistance within pathogens and a decline in the discovery rate of new drugs [40]. We tested it against the most common foodborne pathogens, such as *E. coli*, *L. monocytogenes,* and *S. enterica*. The discovery of antimicrobial peptides in mucus could be up-and-coming, as it could be applied as a preservative in the food industry. In addition, the antibacterial activity of mucus from the body of *H. didactylus* against *P. aeruginosa* is also relevant since this organism is difficult to control with antibiotics due to its high intrinsic resistance [41].

### 2.5. Fractionation by Size Exclusion Chromatography and Peptidomics of the Peptide Fraction

To isolate the most representative peak, which must contain the bioactive peptide fraction to identify the most representative peptides, particularly the fraction containing the most prominent body mucus samples underwent preparative size exclusion chromatography. Due to the limited volume of protein collected, we pooled the samples HdSES1 and HdSES2 (referred to as HdSESP), which previously showed identical molecular size distribution, protein content, and antioxidant activity.

**Figure 3:**
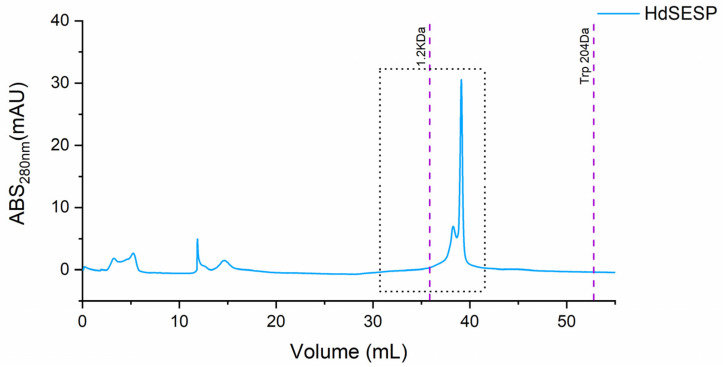
SEC chromatogram of the mucus sample from the HdSESP pooled mucus sample of the HdSES1 individual with the HdSES2 individual.

The purification of the target fraction resulted in a fraction containing a most prominent peak below 1.2 kDa (ca. 800 Da), as illustrated in Figure 3.

Several studies have characterized and demonstrated promising bioactivities of fractions derived from fish mucus. Skin mucus fractions of climbing perch *Anabas testudineus* obtained a bioactive fraction through Sephadex G-25 column [42].

**Figure 4:**
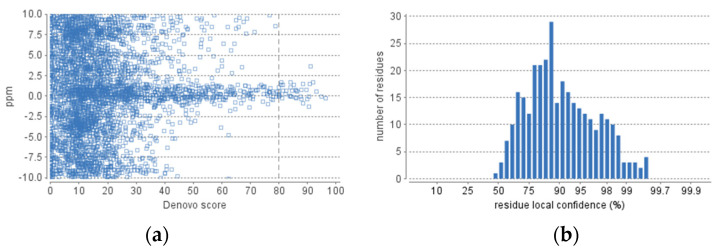
De novo statistics representation. (**a**) Scatterplot of peptide de novo score versus precursor mass error. (**b**) Distribution of residue local confidence in the filtered result.

The obtained fraction of HdSESP, highlighted in Figure 3, was subjected to LC-MS/MS to characterize the sequence of the peptides. Due to the lack of information in proteomic databases for *H. didactylus*, we could not obtain reliable results for peptide identification using database search methods. Consequently, we employed de novo sequencing with the PEAKS Studio© software, considered one of the most reliable tools. The number of MS scans was 25686, with 3329 MS/MS scans. In the analysis process, the number of de novo peptides after the score filter (de novo score ≥ 80) was 37 (Figure 4). The analysis was satisfactory concerning the high degree of confidence in the sequencing.

The analysis using the CAMP_R3_ database yielded the data presented in Table 2. We evaluated the listed peptides using the CAMP_R3_ classifiers (SVM, RF, ANN, and DA) to predict their potential antimicrobial activity. Our analysis categorized some peptides as potentially antimicrobial, while others were not found to exhibit antimicrobial activity (NAMP). These antimicrobial peptides are the most promising candidates for further exploration due to their predicted bioactivity against microorganisms. Among these, the ones that simultaneously offer reasonable estimates of antimicrobial activity for all classifiers are highlighted in Table 2.

We observed diversity of peptide sizes and abundance within the body mucus of *H. didactylus*, as indicated by the identification of peptides with distinct mass and area values. This diversity suggests that multiple antimicrobial compounds contribute to the fish’s defense against pathogens. The identified peptide sequences were subjected to a search of previous reports in the BIOPEP-UWM database, one of the largest databases of bioactive peptides, without finding any match. The results suggest that these peptides may be novel bioactive molecules.

Previous studies have emphasized the antimicrobial properties of specific peptides in fish mucus, such as NK-lysin, CF-14, and hepcidin type 2-like peptides. Li et al. (2019) reported the importance of proline in the middle of the peptide sequence CF-14 for its antimicrobial activity [18]. Similarly, Go et al. (2019) highlighted the antimicrobial properties of peptides with a positively charged structure, such as hepcidin type 2-like, which can disrupt cell membranes by interacting with negatively charged lipopolysaccharides or phospholipids [19]. The peptides we identified in this study share some of these features, particularly the presence of proline in their sequences.

Table 3 ranks the potential antimicrobial peptides based on their bioactivities, determined through PeptideRanker analysis and the BIOPEP-UWM database. PeptideRanker is a server that utilizes a novel N-to-1 neural network to predict bioactive peptides. PeptideRanker assigns a probability of bioactivity to each peptide, with a threshold of 0.5 indicating peptides predicted as bioactive [43]. Among the 15 sequences analyzed, only 7 exhibited potential bioactivity, with VYPFPGPLPN being the most promising candidate.

Using the BIOPEP-UWM database, several peptides displayed a range of potential bioactivities. For instance, DPPNPKNL and DPPNPKLN (highlighted in Table 3) demonstrated significant bioactivity frequency in immunomodulating, neuropeptide, and renin inhibitor categories. Notably, these peptides exhibited inhibitory potential against dipeptidyl peptidase IV (DPP-IV), an enzyme involved in glucose metabolism [44]. The inhibitory properties of these peptides against DPP-IV are particularly interesting, considering the potential of DPP-IV inhibitors in treating type 2 diabetes [44,45].

PAPPPPPP showed a high frequency of bioactive fragments, suggesting its potential involvement in various bioactivities. Our analysis revealed its significant inhibitory activity against ACE and DPP-IV, providing valuable insights into the bioactive repertoire of *H. didactylus* mucus and suggesting potential therapeutic applications in cardiovascular and metabolic health.

In contrast, the peptide EDNSELGQETPTLR exhibited a lower frequency of bioactive fragments than the previously discussed peptides. However, it demonstrated partial inhibitory activity against dipeptidyl peptidase III (DPP-III) and displayed potential immunomodulatory properties. DPP-III inhibitors have been investigated for their potential role in various diseases, including cancer and inflammation [46,47]. The immunomodulatory potential of EDNSELGQETPTLR indicates its potential involvement in regulating the immune response, warranting further investigation for potential therapeutic applications [48].

VYPFPGPLPN, the highest-ranked peptide by PeptideRanker, displayed a high frequency of bioactive fragments, suggesting its potential involvement in multiple bioactivities. Our analysis revealed its potential inhibitory activity against various enzymes, including alpha-glucosidase, DPP-IV, and renin. Alpha-glucosidase inhibitors, similar to DPP-IV inhibitors, have garnered significant interest in managing type 2 diabetes [49], while renin inhibitors have implications in hypertension management [50]. The presence of multiple inhibitory activities in VYPFPGPLPN highlights its potential as a multi-target bioactive peptide.

Additionally, Table 3 highlights that ACE inhibition is the second most frequent bioactivity observed in the identified peptides. Structural characteristics, such as size, chain length, and amino acid sequence type and order, are crucial in determining bioactivity [33]. Previous studies have demonstrated the potent antihypertensive activity of peptides derived from fish hydrolysis, particularly those containing hydrophobic amino acids at the N-terminal and C-terminal ends. For instance, peptides with leucine at the N-terminal and proline at the C-terminal, derived from Alaska pollock *Theragra chalcogramma* skin, exhibited intense antihypertensive activity by inhibiting ACE [29]. Furthermore, peptides rich in proline have been shown to enhance the observed antihypertensive potential in snakehead hydrolysates [33]. These characteristics are also evident in the peptides listed in Table 3, with proline, leucine, and valine present at the terminals.

Overall, this study provides valuable insights into the potential antimicrobial properties of peptides found in the body mucus of *H. didactylus*, as predicted by the CAMP_R3_ database. The results obtained from PeptideRanker and the BIOPEP-UWM database offer valuable information regarding the potential bioactivities of the identified peptides. These findings suggest the presence of diverse and bioactive peptides within the body mucus of *H. didactylus*. Future experimental investigations are necessary to validate the predicted bioactivities [43] and explore these peptides’ specific mechanisms and applications in various therapeutic contexts.

## 3. Materials and Methods

### 3.1. Materials

Angiotensin-I converting enzyme (peptidyl-di-peptidase A, EC 3.4.15.1, 5.1 U/mg), Trolox (6-hydroxy-2,5,7,8-tetramethyl-chroman-2-carboxylic acid), and AAPH [2,20-azobis (2-Int. J. Mol. Sci. 2022, 23, 2439 7 of 9 amidinopropane) dihydrochloride] were obtained from Sigma-Aldrich (St. Louis, MO, USA) and used without further purification. Fluorescein [30,60-dihydroxyspiro (isobenzofuran-1 [3H], 90 [9H]-xanthen)-3-one] was purchased from Fisher Scientific (Hanover Park, IL, USA). The tripeptide Abz-Gly-Phe (NO2)-Pro was obtained from Bachem Feinchemikalien (Bubendorf, Switzerland). Tris [tris (hydroxymethyl) aminomethane] was obtained from Honeywell Fluka (Charlotte, NC, USA). Muller-Hinton Broth was purchased from Biokar Diagnostics (Beauvais, France). Pierce BCA Protein Assay Kit was purchased from Thermo Scientific (Vantaa, Finland).

### 3.2. Mucus Collection

The mucus was collected from *Halobatrachus didactylus* fish captured by fishermen in central west Portugal and later released by our team. Two wild individuals from Sesimbra (HdSES1 and HdSES2) and one captive in our fish facility for more than 3 months was previously captured from Tagus estuary (HdTAG). The dorso-lateral surface was washed with deionized water and the excess liquid was gently removed with a paper towel. A sponge was then used to collect the mucus from the fish skin. No anesthesia or chemical was used during the procedure. The sponges were washed with KPB buffer solution 100 mM and centrifuged to collect the mucus. The mucus samples were stored at −80 °C until further analysis.

### 3.3. Peptide Size Profile

The peptide profile was determined using high-performance size exclusion chromatography (HPSEC). The fish mucus samples were filtered with 0.22 µm filters before HPSEC analysis. An Agilent AdvanceBio SEC column (Agilent Technologies, London, UK), 2.7 µm particle size, 130 Å pore size, and 7.8 inner diameter × 300 mm length was used. The column was eluted isocratically with a phosphate buffer (0.15 M NaH_2_PO_4_ pH 7) at a flow rate of 1 mL/min. The sample injection volume was 10 µL. The instrument used was Waters 2690 with a photodiode array detector (PDA 190–600 nm). The software Empower 3 was used for the data collection. To determine molecular weights of the resulting chromatogram peaks, a calibration curve was made with the following protein standards: Ovalbumin (44,300 Da); Myoglobin (17,600 Da); Cytochrome C (12,327 Da); Aprotinin (6511 Da); Neurotensin (1672 Da); Angiotensin-II (1040 Da); Tyr-Phe dipeptide (328.4 Da); and L-tryptophan (204 Da).

### 3.4. Soluble Protein Content

Bicinchoninic acid (BCA) methodology (Pierce BCA Protein Assay Kit) determined the soluble protein concentration in mucus samples.

### 3.5. Antioxidant Activity

#### 3.5.1. ABTS Assay

The ABTS (2,2-azinobis-(3-ethylbenzothiazoline-6-sulfonic acid)) radical activity is based on an antioxidant’s capacity to scavenge the ABTS’s oxidized state. For this, ABTS radical scavenging activity is performed in a 96-well microplate, following the method described by [51] with some modifications. For the assay, ABTS radical was prepared by the mixture solution of equal parts of ABTS (7 mM) with potassium persulfate (2.44 mM), and then the radical was generated after 16 h. Before performing the assay in the microplate, ABTS radical absorbance was adjusted to 0.70 (±0.02) at 734 nm. In each well, for 20 µL of mucus samples was added 180 µL of ABTS radical. Instead of the sample, ultrapure water was used as control, and seven calibration solutions using Trolox (25–175 µM). The mixture was incubated for 5 min at 30 °C, and the absorbance at 734 nm was measured with a Multidetection plate reader (Synergy H1, Winooski, VT, USA). All measurements were performed in duplicate. The final results were expressed in µmol of Trolox equivalents.

#### 3.5.2. ORAC Assay

The oxygen radical absorbance capacity (ORAC) assay followed the protocol outlined by Coscueta et al. (2019) [52]. A black polystyrene 96-well microplate (Nunc, Roskilde, Denmark) was utilized, and measurements were taken using a Multidetection plate reader (Synergy H1, Vermont, USA) controlled by Gen5 Biotek software version 3.04. Fluorescence was monitored for 80 min at 1 min intervals. Each sample, standard, blank, or control analysis was duplicated. The final ORAC values were expressed as Trolox Equivalents (μmol TE).

### 3.6. Antihypertensive Activity

The angiotensin-converting enzyme-I inhibition (iACE) assay was conducted following the procedure outlined by Coscueta et al. (2019) [52]. The assay was performed using a black polystyrene 96-well microplate (Nunc, Denmark), and fluorescence was monitored at 1 min intervals for 80 min using a Multidetection plate reader (Synergy H1, Vermont, USA) controlled by Gen5 Biotek software version 3.04. Each sample, blank, and control analysis was carried out in triplicate. The iACE was quantified as the concentration required to inhibit 50% of the enzymatic activity (IC_50_). The IC_50_ values were determined using non-linear modelling of the data obtained.

### 3.7. Antimicrobial Activity

The antimicrobial activity of the mucus samples was evaluated following the method by [53] against the following pathogenic bacteria: Gram-negative *Escherichia coli* ATCC 25922, *Salmonella enterica serovar Enteritidis* ATCC 13076, and *Pseudomonas aeruginosa* ATCC 27853, and Gram-positive *Listeria monocytogenes* NCTC 10357. The mucus samples were combined with a 1/10 ratio of inoculum containing 1% (*v/v*) of bacteria cultured for 24 h in Mueller–Hinton Broth (MHB). For the positive control, deionized water was used instead of mucus samples with a 1% inoculum, while deionized water with MHB served as the negative control. The mixture was then transferred to a 96-well microplate (Sarstedt, Germany), and the optical density (OD) at 600 nm was measured every hour for a 24 h period at 37 °C using a microplate reader (Multiskan GO, Thermo Scientific, Finland). An increase in OD indicated bacterial growth.

### 3.8. Preparative Size Exclusion Chromatography

The mucus samples from HdTAG and HDSESP (pooled from HDSES1 with HDSES2) were loaded onto two size exclusion chromatography (SEC) columns, namely Superdex Peptide 10/300 GL and Superdex 200 Increase 10/300 GL, connected in series, using an ÄKTA pure chromatography system. Ultrapure water was used as the eluent for the columns, and the peaks were detected at an absorbance of 280 nm. The elution flow rate was set at 0.5 mL/min, and fractions of 5 mL were collected starting from the time of sample loading on the column. Two fractions containing the most intense peaks were pooled.

### 3.9. Peptidomic Analysis

Peptide identification and quantification were conducted using nanoLC-MS/MS, following the method described by Osório et al. (2021) [54]. Tandem mass spectra were processed with PEAKS Studio version 10.6 (Bioinformatics Solutions Inc., Waterloo, ON, Canada). The DENOVO analysis was configured assuming no digestion enzyme, with a fragment ion mass tolerance of 0.02 Da, a parent ion tolerance of 10.0 ppm, and a maximum of 3 variable PTMs per peptide.

The identified peptide sequences were categorized based on their potential antimicrobial activity, filtering out those not expected to possess such activity. To predict the bioactivity in silico, all identified peptides were analyzed using the CAMP_R3_ (Collection of Antimicrobial Peptides) [55] with Support Vector Machine (SVM) classifier, Random Forest (RF) classifier, Artificial Neural Network (ANN) classifier, and Discriminant Analysis classifier.

The potentially antimicrobial sequences were further analyzed to estimate their overall bioactive potential. For in silico bioactivity prediction of each peptide, a ranking was initially performed using PeptideRanker [56], a server that predicts bioactive peptides based on a novel N-to-1 neural network [57]. Subsequently, all these peptides were analyzed using the BIOPEP-UWM database [58] to estimate potential bioactivities [59,60].

### 3.10. Statistical Analysis

Statistical analysis was performed using RStudio v2023.06.0. The mean values obtained from two replicates were subjected to analysis of variance (ANOVA). Mean separation was determined using Tukey’s post hoc test at a 5% significance level. Homoscedasticity of variance was assessed using Cochran’s and Bartlett’s tests at a 5% significance level.

## 4. Conclusions

In conclusion, our study provides the first peptide profile for the Lusitanian toadfish *Halobatrachus didactylus* epidermal mucus. We also describe its antioxidant activity, antihypertensive activity, antimicrobial activity of *H. didactylus*’s body mucus, and the peptidomics obtained for the most representative chromatographic peak. The peptide profiles revealed the presence of bioactive peptides with smaller molecular sizes, indicating their potential role in various biological activities. The body mucus exhibited significant antioxidant activity, surpassing values reported for other fish species. The inhibitory activity against ACE highlighted the potential of *H. didactylus* mucus as an alternative source of antihypertensive peptides.

Moreover, the mucus displayed antimicrobial solid effects against pathogenic bacteria, expanding the understanding of its biological defense mechanisms. The fractionation and peptidomics analysis of the mucus led to identifying novel peptides with bioactive properties estimated in silico. Further in vitro assays and cytotoxicity tests are required to validate the potential applications of these peptides in the pharmaceutical and food industries. Overall, this research contributes to exploring and valorizing the properties of toxic *H. didactylus* epidermal mucus and highlighting the potential of fish ichthyocrinotoxins that remain a largely untapped source of bioactive molecules.

## 5. Patents

New peptides and uses thereof. Instituto Nacional da Propriedade Industrial. Patent Nº.: PT118365. 5 December 2022.

## Figures and Tables

**Table 1:** Protein concentration (BCA) and antioxidant (ORAC and ABTS) activity of mucus samples from *H. didactylus* individuals.

Sample	Protein (µg BSA/mL)	ORAC (µmol TE/g Mucus Protein)	ABTS (µmol TE/g Mucus Protein)
HdTAG	13,260 ± 342 ^a^	2371 ± 97 ^a^	112 ± 3 ^a^
HdSES1	344 ± 4 ^b^	164 ± 28 ^b^	154 ± 6 ^b^
HdSES2	447 ± 98 ^b^	188 ± 6 ^b^	129 ± 4 ^a^

All values are mean (n = 2) ± SD. ^a,b^ The different letter superscripts in the same column indicate that the values differed significantly (*p <* 0.05).

**Table 2:** Peptide classification based on antimicrobial potential predicted in silico.

Mass (Da)	Area	Seq. ^1^	Class ^2,3^			
			SVM	RF	ANN	DA
1589.8	2.89 × 10^8^	EDNSELGQETPTLR	AMP	NAMP	NAMP	NAMP
1101.6	1.26 × 10^8^	VYPFPGPLPN	NAMP	NAMP	AMP	NAMP
839.5	9.33 × 10^7^	PFPGPLPN	NAMP	NAMP	AMP	NAMP
839.5	9.33 × 10^7^	PFGPPLPN	NAMP	NAMP	AMP	NAMP
889.5	7.54 × 10^7^	EVGDEVLK	NAMP	NAMP	NAMP	NAMP
887.5	5.67 × 10^7^	ELGDEVPK	NAMP	NAMP	AMP	NAMP
960.5	4.81 × 10^7^	AEVGDEVLK	NAMP	NAMP	NAMP	NAMP
760.4	2.89 × 10^7^	VGDEVKL	NAMP	NAMP	NAMP	NAMP
760.4	2.89 × 10^7^	VGDEVLK	NAMP	NAMP	NAMP	NAMP
1015.6	2.40 × 10^7^	QETPTLRVA	AMP	NAMP	NAMP	NAMP
1101.6	2.33 × 10^7^	DDVGDKELLP	NAMP	NAMP	NAMP	NAMP
785.5	2.28 × 10^7^	EVVLVEP	AMP	NAMP	NAMP	NAMP
1086.6	2.12 × 10^7^	QAELGDEVPK	NAMP	NAMP	NAMP	NAMP
895.5	1.59 × 10^7^	DPPNPKNL	AMP	AMP	AMP	AMP
895.5	1.59 × 10^7^	DPPNPKLN	AMP	AMP	AMP	AMP
1474.7	1.44 × 10^7^	APAGATDAKDEDLVT	NAMP	NAMP	NAMP	NAMP
887.4	6.53 × 10^6^	TDPPELNT	AMP	NAMP	NAMP	NAMP
950.5	4.19 × 10^6^	VAPPNPQNL	NAMP	NAMP	NAMP	NAMP
1026.6	4.13 × 10^6^	PPFLQVVPE	NAMP	NAMP	NAMP	NAMP
993.5	3.87 × 10^6^	PLVNHEGAGV	NAMP	NAMP	NAMP	NAMP
985.4	3.41 × 10^6^	DGGPPSPDNE	NAMP	AMP	NAMP	NAMP
791.4	3.04 × 10^6^	PFYPGPL	NAMP	NAMP	AMP	NAMP
770.4	2.05 × 10^6^	PAPPPPPP	AMP	AMP	NAMP	NAMP
845.5	6.27 × 10^5^	QETPTLR	AMP	NAMP	NAMP	NAMP
844.5	3.56 × 10^5^	NPATVLKT	NAMP	NAMP	AMP	NAMP
832.5	1.02 × 10^5^	LTKVLEE	NAMP	NAMP	NAMP	NAMP

^1^ The peptides are arranged in descending order of abundance, as indicated by the “Area” column. ^2^ AMP denotes peptides classified as potentially antimicrobial, while NAMP denotes peptides not exhibiting antimicrobial activity. ^3^ SVM, RF, ANN, and DA represent the classification results obtained from CAMP_R3_ classifiers. Peptides that simultaneously offer reasonable estimates of antimicrobial activity for all classifiers are highlighted in bold (for other criteria, see Table 3 below).

**Table 3:** Peptide classification based on potential bioactivity predicted in silico.

Seq. ^1^	PeptideRanker ^2^	BIOPEP (A) ^3^															
		ACE inhibitor	Alpha-glucosidase Inhibitor	Antiamnestic	Antioxidative	Antithrombotic	Chemotactic	Dipeptidyl Peptidase III Inhibitor	Dipeptidyl Peptidase IV Inhibitor	Immunomodulating	Inhibitor	Neuropeptide	Opioid Agonist	Opioid	Regulating	Renin Inhibitor	Stimulating
DPPNPKNL	0.73075	0.125	0.125						0.750								
DPPNPKLN	0.68444	0.375	0.125						0.750								
PAPPPPPP	0.96263	1.375	0.625						1.250								
EDNSELGQETPTLR	0.04606	0.357			0.071			0.071	0.429			0.071				0.071	0.071
VYPFPGPLPN	0.96638	1.200	0.100	0.300	0.100	0.300	0.100	0.200	0.900	0.200	0.100		0.100	0.200	0.300		
PFPGPLPN	0.91394	1.000		0.375		0.375	0.125	0.125	0.875		0.125				0.375		
PFGPPLPN	0.91526	1.000	0.125	0.125	0.125	0.125		0.125	0.875		0.125				0.125		
ELGDEVPK	0.19243	0.625			0.125				0.375								
QETPTLRVA	0.19192	0.333	0.111					0.222	0.667							0.111	
EVVLVEP	0.18655	0.571	0.143						0.857								0.286
TDPPELNT	0.09808	0.250	0.250		0.250			0.250	0.625								
DGGPPSPDNE	0.37634	0.500	0.100	0.100	0.100	0.100			0.700		0.100				0.100		
PFYPGPL	0.11515	1.000	0.286	0.429		0.429	0.143	0.143	0.714		0.143				0.429		
QETPTLR	0.78508	0.429	0.143					0.143	0.714							0.143	
NPATVLKT	0.14493				0.125				0.750								0.125

^1^ The peptides are arranged in descending order based on their potential antimicrobial prediction by CAMP_R3_, with secondary order given to their abundance. ^2^ PeptideRanker scores indicate the probability of the peptide being bioactive. ^3^ The “A” represents the frequency of bioactive fragments in a protein, categorized by various bioactivity types predicted by BIOPEP-UWM.

## Data Availability

The data presented in this study are available on request from the corresponding author. The data are not publicly available as the patenting process is still ongoing at the time this article is submitted.

## References

[1] Uddin T.M., Chakraborty A.J., Khusro A., Zidan B.R.M., Mitra S., Emran T.B., Dhama K., Ripon M.K.H., Gajdács M., Sahibzada M.U.K. (2021). Antibiotic Resistance in Microbes: History, Mechanisms, Therapeutic Strategies and Future Prospects. J. Infect. Public Health.

[2] Ilan Y. (2022). Next-Generation Personalized Medicine: Implementation of Variability Patterns for Overcoming Drug Resistance in Chronic Diseases. J. Pers. Med..

[3] Durand G.A., Raoult D., Dubourg G. (2019). Antibiotic Discovery: History, Methods and Perspectives. Int. J. Antimicrob. Agents.

[4] Du Z., Comer J., Li Y. (2023). Bioinformatics Approaches to Discovering Food-Derived Bioactive Peptides: Reviews and Perspectives. TrAC Trends Anal. Chem..

[5] Grosberg R.K., Vermeij G.J., Wainwright P.C. (2012). Biodiversity in Water and on Land. Curr. Biol..

[6] Cragg G.M., Newman D.J. (2013). Natural Products: A Continuing Source of Novel Drug Leads. Biochim. Biophys. Acta Gen. Subj..

[7] Kim S.K., Wijesekara I. (2010). Development and Biological Activities of Marine-Derived Bioactive Peptides: A Review. J. Funct. Foods.

[8] Smith W.L., Wheeler W.C. (2006). Venom Evolution Widespread in Fishes: A Phylogenetic Road Map for the Bioprospecting of Piscine Venoms. J. Hered..

[9] Rajanbabu V., Chen J.Y. (2011). Applications of Antimicrobial Peptides from Fish and Perspectives for the Future. Peptides.

[10] Sridhar A., Manikandan D.B., Marimuthu S.K., Murugesan M., Ramasamy T. (2021). Methanol Skin Mucus Extract of Mrigal (*Cirrhinus mrigala*) Fish Peptide Targeting Viral Particles of Infectious Pancreatic Necrosis Virus (IPNV) and Infectious Salmon Anemia Virus (ISAV): An in Silico Approach. Int. J. Pept. Res. Ther..

[11] Ziegman R., Alewood P. (2015). Bioactive Components in Fish Venoms. Toxins.

[12] Qadiri S.S.N., Makesh M., Rajendran K.V., Rathore G., Purushothaman C.S. (2019). Specific Immune Response in Mucosal and Systemic Compartments of *Cirrhinus mrigala* Vaccinated against *Edwardsiella tarda*: In Vivo Kinetics Using Different Antigen Delivery Routes. J. World Aquac. Soc..

[13] Hoseinifar S.H., Jahazi M.A., Nikdehghan N., Van Doan H., Volpe M.G., Paolucci M. (2020). Effects of Dietary Polyphenols from Agricultural By-Products on Mucosal and Humoral Immune and Antioxidant Responses of Convict Cichlid (*Amatitlania nigrofasciata*). Aquaculture.

[14] Heydari M., Firouzbakhsh F., Paknejad H. (2020). Effects of *Mentha longifolia* Extract on Some Blood and Immune Parameters, and Disease Resistance against Yersiniosis in Rainbow Trout. Aquaculture.

[15] Mohammadi G., Adorian T.J., Rafiee G. (2020). Beneficial Effects of *Bacillus subtilis* on Water Quality, Growth, Immune Responses, Endotoxemia and Protection against Lipopolysaccharide-Induced Damages in *Oreochromis niloticus* under Biofloc Technology System. Aquac. Nutr..

[16] Oliveira M., Tvarijonaviciute A., Trindade T., Soares A., Tort L., Teles M. (2018). Can Non-Invasive Methods Be Used to Assess Effects of Nanoparticles in Fish?. Ecol. Indic..

[17] Valero Y., Cortés J., Mercado L. (2019). NK-Lysin from Skin-Secreted Mucus of Atlantic Salmon and Its Potential Role in Bacteriostatic Activity. Fish Shellfish Immunol..

[18] Li T., Liu Q., Wang D., Li J. (2019). Characterization and Antimicrobial Mechanism of CF-14, a New Antimicrobial Peptide from the Epidermal Mucus of Catfish. Fish Shellfish Immunol..

[19] Go H.-J., Kim C.-H., Park J.B., Kim T.Y., Lee T.K., Oh H.Y., Park N.G. (2019). Biochemical and Molecular Identification of a Novel Hepcidin Type 2-like Antimicrobial Peptide in the Skin Mucus of the Pufferfish *Takifugu pardalis*. Fish Shellfish Immunol..

[20] Lopes-Ferreira M., Barbaro K.C., Cardoso D.F., Moura-Da-Silva A.M., Mota I. (1998). *Thalassophryne nattereri* Fish Venom: Biological and Biochemical Characterixation and Serum Neutralization of Its Toxic Activities. Toxicon.

[21] Magalhães G.S., Lopes-Ferreira M., Junqueira-De-Azevedo I.L.M., Spencer P.J., Araújo M.S., Portaro F.C.V., Ma L., Valente R.H., Juliano L., Fox J.W. (2005). Natterins, a New Class of Proteins with Kininogenase Activity Characterized from *Thalassophryne nattereri* Fish Venom. Biochimie.

[22] Nair M.S.R., Leong I., Nayar M.S.B. (1982). Ichthyotoxins from the Oyster Toadfish, *Opsanus tau* (Linnaeus). Toxicon.

[23] Marques J.F., Santos M.J., Costa J.L., Costa M.J., Cabral H.N. (2005). Metazoan Parasites as Biological Indicators of Population Structure of *Halobatrachus didactylus* on the Portuguese Coast. J. Appl. Ichthyol..

[24] Cotter J.C., Pereira T.J., Costa M.J., Costa J.L. (2013). Distribution, Abundance, Population Structure and Activity of *Halobatrachus didactylus* in the Tagus Estuary (Portugal) and Adjacent Coastal Waters. J. Mar. Biol. Assoc. UK.

[25] Huang D., Boxin O.U., Prior R.L. (2005). The Chemistry behind Antioxidant Capacity Assays. J. Agric. Food Chem..

[26] Tagami M., Kuwahara J. (2020). Evaluation of Antioxidant Activity and Amino Acids in the Mucus of Mackerel for Cosmetic Applications. J. Oleo Sci..

[27] Wen C., Zhang J., Zhang H., Duan Y., Ma H. (2020). Plant Protein-Derived Antioxidant Peptides: Isolation, Identification, Mechanism of Action and Application in Food Systems: A Review. Trends Food Sci. Technol..

[28] Coscueta E.R., Brassesco M.E., Pintado M. (2021). Collagen-Based Bioactive Bromelain Hydrolysate from Salt-Cured Cod Skin. Appl. Sci..

[29] Kim S.K., Ngo D.H., Vo T.S. (2012). Marine Fish-Derived Bioactive Peptides as Potential Antihypertensive Agents.

[30] Abdelhedi O., Nasri M. (2019). Basic and Recent Advances in Marine Antihypertensive Peptides: Production, Structure-Activity Relationship and Bioavailability. Trends Food Sci. Technol..

[31] Yathisha U.G., Bhat I., Karunasagar I., Mamatha B.S. (2019). Antihypertensive Activity of Fish Protein Hydrolysates and Its Peptides. Crit. Rev. Food Sci. Nutr..

[32] Abdel-Hamid M., Otte J., De Gobba C., Osman A., Hamad E. (2017). Angiotensin I-Converting Enzyme Inhibitory Activity and Antioxidant Capacity of Bioactive Peptides Derived from Enzymatic Hydrolysis of Buffalo Milk Proteins. Int. Dairy J..

[33] Abachi S., Bazinet L., Beaulieu L. (2019). Antihypertensive and Angiotensin-i-Converting Enzyme (ACE)-Inhibitory Peptides from Fish as Potential Cardioprotective Compounds. Mar. Drugs.

[34] Kumar P., Rajeshwaran T., Priya P., Kailasam M., Biswas G., Ghoshal T.K., Vijayan K.K., Arasu A.R.T. (2019). Comparative Immunological and Biochemical Properties of the Epidermal Mucus from Three Brackishwater Fishes. Proc. Natl. Acad. Sci. India Sect. B Biol. Sci..

[35] Guluarte C., Reyes-Becerril M., Gonzalez-Silvera D., Cuesta A., Angulo C., Esteban M.Á. (2019). Probiotic Properties and Fatty Acid Composition of the Yeast *Kluyveromyces lactis* M3. In Vivo Immunomodulatory Activities in Gilthead Seabream (*Sparus Aurata*). Fish Shellfish Immunol..

[36] Soltanian S., Gholamhosseini A. (2019). The Effects of Starvation on Some Epidermal Mucus Immune Parameters in Rainbow Trout, Oncorhynchus Mykiss. Int. J. Aquat. Biol..

[37] Abdel-Shafi S., Osman A., Al-Mohammadi A.-R., Enan G., Kamal N., Sitohy M. (2019). Biochemical, Biological Characteristics and Antibacterial Activity of Glycoprotein Extracted from the Epidermal Mucus of African Catfish (*Clarias gariepinus*). Int. J. Biol. Macromol..

[38] Patel M., Ashraf M.S., Siddiqui A.J., Ashraf S.A., Sachidanandan M., Snoussi M., Adnan M., Hadi S. (2020). Profiling and Role of Bioactive Molecules from *Puntius sophore* (Freshwater/Brackish Fish) Skin Mucus with Its Potent Antibacterial, Antiadhesion, and Antibiofilm Activities. Biomolecules.

[39] Fuochi V., Li Volti G., Camiolo G., Tiralongo F., Giallongo C., Distefano A., Petronio G.P., Barbagallo I., Viola M., Furneri P.M. (2017). Antimicrobial and Anti-Proliferative Effects of Skin Mucus Derived from *Dasyatis pastinaca* (Linnaeus, 1758). Mar. Drugs.

[40] Cantisani M., Finamore E., Mignogna E., Falanga A., Nicoletti G.F., Pedone C., Morelli G., Leone M., Galdiero M., Galdiero S. (2014). Structural Insights into and Activity Analysis of the Antimicrobial Peptide Myxinidin. Antimicrob. Agents Chemother..

[41] Jiang Z., Vasil A.I., Gera L., Vasil M.L., Hodges R.S. (2011). Rational Design of α-Helical Antimicrobial Peptides to Target Gram-Negative Pathogens, Acinetobacter Baumannii and Pseudomonas Aeruginosa: Utilization of Charge, “Specificity Determinants,” Total Hydrophobicity, Hydrophobe Type and Location as Design Parameters to Improve the Therapeutic Ratio. Chem. Biol. Drug Des..

[42] Najm A.A.K., Azfaralariff A., Dyari H.R.E., Othman B.A., Shahid M., Khalili N., Law D., Syed Alwi S.S., Fazry S. (2021). Anti-Breast Cancer Synthetic Peptides Derived from the Anabas Testudineus Skin Mucus Fractions. Sci. Rep..

[43] Coscueta E.R., Batista P., Gomes J.E.G., da Silva R., Pintado M.M. (2022). Screening of Novel Bioactive Peptides from Goat Casein: In Silico to In Vitro Validation. Int. J. Mol. Sci..

[44] Ma C., Liu D., Hao H., Wu X. (2022). Identification of the DPP-IV Inhibitory Peptides from Donkey Blood and Regulatory Effect on the Gut Microbiota of Type 2 Diabetic Mice. Foods.

[45] Nongonierma A.B., Fitzgerald R.J. (2014). An in Silico Model to Predict the Potential of Dietary Proteins as Sources of Dipeptidyl Peptidase IV (DPP-IV) Inhibitory Peptides. Food Chem..

[46] Prajapati S.C., Chauhan S.S. (2011). Dipeptidyl Peptidase III: A Multifaceted Oligopeptide N-End Cutter. FEBS J..

[47] Abramić M., Agić D. (2022). Survey of Dipeptidyl Peptidase III Inhibitors: From Small Molecules of Microbial or Synthetic Origin to Aprotinin. Molecules.

[48] Kang H.K., Lee H.H., Seo C.H., Park Y. (2019). Antimicrobial and Immunomodulatory Properties and Applications of Marine-Derived Proteins and Peptides. Mar. Drugs.

[49] Dirir A.M., Daou M., Yousef A.F., Yousef L.F. (2022). A Review of Alpha-Glucosidase Inhibitors from Plants as Potential Candidates for the Treatment of Type-2 Diabetes. Phytochem. Rev..

[50] Massolini B.D., Contieri S.S.G., Lazarini G.S., Bellacosa P.A., Dobre M., Petroianu G., Brateanu A., Campos L.A., Baltatu O.C. (2020). Therapeutic Renin Inhibition in Diabetic Nephropathy—A Review of the Physiological Evidence. Front. Physiol..

[51] Gonçalves B., Falco V., Moutinho-Pereira J., Bacelar E., Peixoto F., Correia C. (2009). Effects of Elevated CO2 on Grapevine (*Vitis vinifera* L.): Volatile Composition, Phenolic Content, and in Vitro Antioxidant Activity of Red Wine. J. Agric. Food Chem..

[52] Coscueta E.R., Campos D.A., Osório H., Nerli B.B., Pintado M. (2019). Enzymatic Soy Protein Hydrolysis: A Tool for Biofunctional Food Ingredient Production. Food Chem. X.

[53] Alexandre E.M.C., Silva S., Santos S.A.O., Silvestre A.J.D., Duarte M.F., Saraiva J.A., Pintado M. (2019). Antimicrobial Activity of Pomegranate Peel Extracts Performed by High Pressure and Enzymatic Assisted Extraction. Food Res. Int..

[54] Osório H., Silva C., Ferreira M., Gullo I., Máximo V., Barros R., Mendonça F., Oliveira C., Carneiro F. (2021). Proteomics Analysis of Gastric Cancer Patients with Diabetes Mellitus. J. Clin. Med..

[55] CAMP: Collection of Sequences and Structures of Antimicrobial Peptides. http://www.camp3.bicnirrh.res.in/.

[56] PeptideRanker. http://distilldeep.ucd.ie/PeptideRanker/.

[57] Mooney C., Haslam N.J., Pollastri G., Shields D.C. (2012). Towards the Improved Discovery and Design of Functional Peptides: Common Features of Diverse Classes Permit Generalized Prediction of Bioactivity. PLoS ONE.

[58] No BIOPEP-UWM—Katedra Biochemii Zywno’sci. https://biochemia.uwm.edu.pl/biopep-uwm/.

[59] Minkiewicz P., Iwaniak A., Darewicz M. (2019). BIOPEP-UWM Database of Bioactive Peptides: Current Opportunities. Int. J. Mol. Sci..

[60] Minkiewicz P., Dziuba J., Iwaniak A., Dziuba M., Darewicz M. (2008). BIOPEP Database and Other Programs for Processing Bioactive Peptide Sequences. J. AOAC Int..

